# Computer Assisted Pesticide and PCB Identification System (CAPPIS)

**DOI:** 10.6028/jres.093.052

**Published:** 1988-06-01

**Authors:** Joseph E. Dierkes

**Affiliations:** New Jersey State Department of Health, John Fitch Plaza CN 361, Trenton, NJ 08625

Environmental samples (water, air, soil, etc.) are routinely analyzed in the state laboratory for pesticide and PCB residues using gas chromatographs (GCs) equipped with electron capture detectors (ECDs), and packed glass (4 mm × 2 m) columns of differing polarity. In a typical analysis, one primary column (1.5% OV-17/1.95% OV-210) is used to quantitate results obtained after confirmation by a secondary column (4% SE-30/6% OV-210) and occasionally a third column (5% OV-210, 3% OV- 1, etc.).

Two standard mixtures of pesticides are injected before any samples are analyzed. After verifying retention times (RTs) for key pesticides (i.e., pp′- DDT) in these standards, actual sample analysis may begin. Should any peaks be encountered, then their RTs are compared with those of the standards. If the sample peak RT on each column used matches the RT of the pesticide standard on each column within certain limits, identification is tentatively confirmed. If a multicomponent residue such as chlordane, toxaphene, or a PCB is found, then the appropriate or suspected standard is also injected and chromatographed. Each sample chromatogram is checked for the presence and proper RT of any surrogate compounds (Mirex or dibutylchlorendate) used.

The peak height ratios between the columns are next examined for a similar ratio obtained for that of the pesticide and the standard, thus ruling out false identifications due to interferences. Following U.S.E.P.A. practice, positive matches are quantitated by a comparison (peak height or area) with known concentrations of standards, as long as the peak heights between sample and standards are within 25% of each other. If the positive result is the surrogate compound, then its percent recovery is also calculated.

It was desired to develop a computer algorithm to perform all these checks and measurements with greater accuracy and speed, provide a considerable amount of quality control documentation for large numbers of samples, and at the same time meet or surpass stringent U.S.E.P.A. and IFB analytical requirements.

To this end, a battery of GCs was interfaced with a mainframe computer system. Sample chromatograms are produced by standard 1 mV recorders and the GC signals are simultaneously digitized by analog-to-digital converters. The digitized data from each analysis are arranged into a definite set of records in a file by a modified post run program which assigns the file a unique number and stores it on the disk. Any chromatogram can be called up at a later time for access by the software package, designated CAPPIS, by referring to its file number.

This package consists of a set of simple BASIC/FORTRAN programs which could be modified to work with various analytical systems. All that would be needed would be to set up unique digitized data files and define the ways the software could access them.

After a GC is set up, its operating parameters (column types, carrier gas type and flow rate, temperature, etc.) are stored on the disk in a file generated by one of these programs. Next, retention time windows are generated by another program and also stored on disk. This completes the initial setup, and is essentially done once. Any later changes in the operating parameters, columns, or windows are very easily accomplished by rerunning one or both of these programs.

When a series of samples is injected and analyzed, all chromatograms thus generated are labeled with sample identifications, sizes, weights, volume injected, and attenuation used with yet another program. Then, the user is ready to invoke CAPPIS to analyze the data.

This analytical package consists of a menu from which the user chooses one of [currently] three options:

1. identify and quantitate pesticide residues with two or with three columns;
2. quantitate a multicomponent sample such as a PCB, chlordane, or toxaphene, when the identity of the sample has been established;
3. compare different multicomponent standards with a multicomponent sample to deduce its identity.

Essentially, the software sorts the peak data into various sets of arrays which are then manipulated according to which option is chosen. For the first option, it compares sample chromatograms with those of different pesticide standards analyzed the same day. Positive identifications are based upon retention time matches between all the columns used as long as the RT for each peak in question is within its specified window. After passing a peak height ratio test, a result is calculated; if it is above experimentally determined detection limits, it is printed on the report.

Since it is not practical to maintain any sort of “library” of PCB patterns (due to varying detector response, operating parameters and column characteristics between different instruments), option #3 functions as a useful tool in assigning an identification between similar PCBs (e.g., Ar1248 vs Ar1254). Once the identity of a particular PCD has been established, it is a simple matter to quantitate the results with option #2.

In contrast to the manual quantitation method, the sum of the peak heights from ALL the columns used is employed for computing results, not simply the peak height from the primary column. When quantitating a set of multicomponent samples versus a standard, CAPPIS adjusts itself to varying numbers of peaks, so that the maximum possible number of peak matches can be used in each case. This provides greater accuracy and reproducibility due to an error leveling effect.

The software is open-ended. By adding additional testing loops either internally or in the form of subroutines, further refinements, currently under study in our laboratory, can be made to option #3. Peak data arrays can be extended in various directions to encompass the use of three or more different standards and/or columns. Arrays could also be added to option #1 for additional testing loops such as relative retention time ratios for deducing possible identifications of pesticides not contained in the standards.

During the past several months that CAPPIS has been in routine use, a time savings of one man-hour per instrument per day has been realized. The software has been used to successfully pass proficiency tests at the federal and state levels. Additionally, it has provided a reliable method of tracking both instrument performance and quality control trends.

